# Investigation and management of childhood stroke

**DOI:** 10.1016/j.paed.2010.08.005

**Published:** 2010-09

**Authors:** Fenella Kirkham

**Affiliations:** **Fenella Kirkham MD FRCPCH** is a Professor in Paediatric Neurology at the Neurosciences Unit, University College London, Institute of Child Health, 30 Guilford Street, London WC1N 1EH and Southampton General Hospital, Southampton SO16 6YD, UK

**Keywords:** anaemia, cerebral venous sinus thrombosis, cerebrovascular diseases, magnetic resonance, posterior reversible encephalopathy syndrome, vasculopathy

## Abstract

It is now clear that a number of paediatric emergencies with a neurological presentation, including hemiparesis, visual loss, seizures and coma, commonly have a vascular basis which may not be obvious on CT scan. Although many children do well, as there is significant mortality as well as morbidity for childhood stroke, in addition to a high risk for recurrence, making a diagnosis in the acute phase important. Venography and arteriography (including the neck vessels if the intracranial vessels are normal) are usually indicated despite the problems i.e. contrast CT requires a high dose of radiation while emergency MR usually requires anaesthesia and conventional arteriography carries a small risk of stroke. Surgical decompression may be life-saving in ischaemic as well as haemorrhagic stroke. It is unusual for children with anterior circulation stroke to be triaged quickly enough (<4.5 h) for thrombolysis but this may occasionally be appropriate in posterior circulation occlusion associated with coma, where the time window is longer (<12 h). Anticoagulation carries relatively low risk and may be of benefit for children with venous sinus thrombosis (acutely and when at risk subsequently) or extracranial dissection. Aspirin to attempt to reduce the recurrence risk is appropriate in the medium term for the majority of patients with arterial ischaemic stroke. Iron and B vitamin deficiencies should be excluded or treated.

## Introduction

Stroke remains one of the commonest causes of death and disability in childhood. The last few years have seen advances in our understanding of the pathology because of the improvements in non invasive methods of imaging the cerebrovascular circulation. There have been controversies about optimal timing for investigation and in particular about emergency management strategies but a consensus is now emerging. This article outlines the results from the recent studies and suggests an evidence based pathway for investigation and management. More details can be found in the Royal College of Physicians (UK), American Stroke Association and Chest guidelines (see Further reading).

## Epidemiology

Stroke affects between 1.3 and 13 per 100,000 children/year; at least a third are haemorrhagic while a similar proportion have ischaemic stroke. At a practical level that means that at least 300 children in the United Kingdom have a stroke every year. In childhood there are also a large number of stroke mimics which can account for up to one third of the diagnosed cases and which are very important to exclude before treatment strategies are considered. Fatalities are highest for those with haemorrhagic stroke, mainly related to the increased intracranial pressure associated with space-occupying lesions such as intracranial haematomata, but some children with arterial ischaemic stroke also die of brain swelling. Mortality and morbidity are certainly higher for those who already have an underlying condition such as cardiac malformation or sickle cell disease and this group of patients is one where there has been increasing interest recently in making the diagnosis. The proportion of acutely ill children, for example in intensive care, that have had a stroke is doubled if radiological reports are included in addition to clinical coding. There should certainly be a low threshold for vascular imaging in children who are acutely sick and have any neurological complications including seizures as well as hemiparesis.

## Pathology

One study from the middle of the 20th century found that nearly 9% of children who died and had a post mortem in a single centre had a vascular diagnosis, with over half of these patients having had venous sinus thrombosis, including a substantial proportion of those with congenital heart disease. Dissection and occlusion of the internal carotid and basilar system were also documented, while patients dying with stroke in the context of Sickle Cell Disease appeared to have severe intracranial cerebrovascular disease or extensive white matter abnormalities. These autopsy studies are likely to have been biased, however, and there are relatively few data from patients with conditions such as post varicella arteriopathy, which the patient usually survives.

## Clinical clues to the diagnosis (Table 1)

Taking a history is very important particularly of any underlying condition and of recent trauma or infections, including chickenpox within the previous year and apparently minor upper respiratory tract infections. Family history is also important and should be documented for every first degree relative to determine whether there is any history of early stroke, coronary heart disease or venous thrombosis, for example in the legs. Pregnancy and birth history are important in establishing whether the child’s mother had hypertension and also because birth weight appears to be lower in children with arterial ischaemic stroke compared with normative data.

In terms of the physical signs a careful look for neurocutaneous stigmata is important, as otherwise discounted lesions such as linear sebaceous naevus or scleroderma ‘en-coup-de-sabre’ may be associated with cerebrovascular disease.

## Investigations (Table 1)

### Neuroimaging

**Figure 1:**
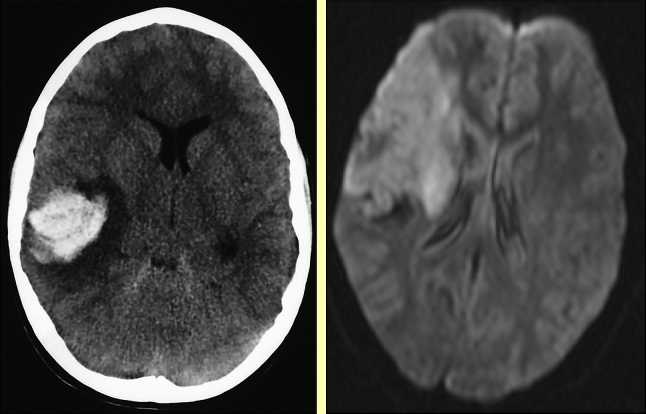
Emergency imaging in unilateral stroke. Left: CT showing acute intracerebral haemorrhage with surrounding focal oedema in a child presenting in coma after a focal seizure, Right: MRI showing infarction in the territory of the occluded middle cerebral artery in a child with a dense hemiparesis; the CT scan had been reported as normal although there was subtle abnormality.

**Figure 2:**
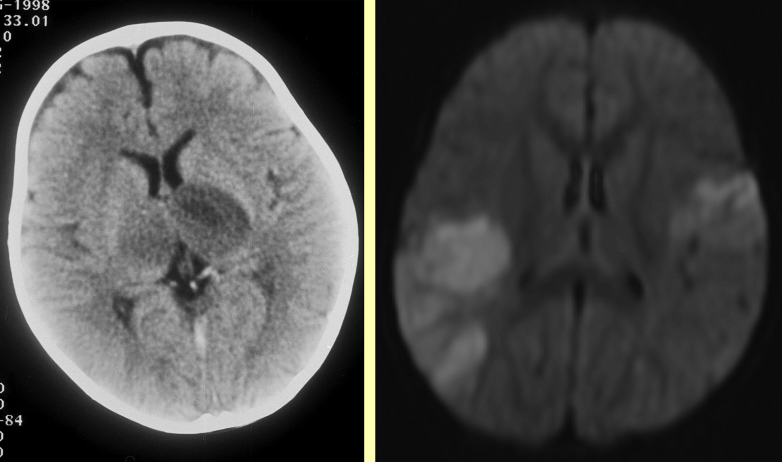
Emergency imaging in bilateral stroke. Left: thalamic infarction in a child with iron deficiency anaemia and venous sinus thrombosis, Right: MRI showing bilateral infarction in a child with acute myeloid leukaemia.

**Figure 3:**
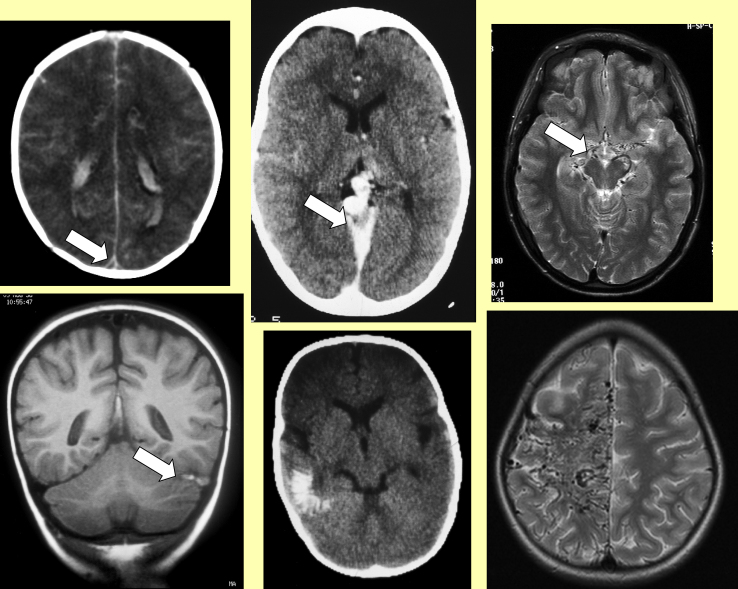
Vascular abnormalities seen on parenchymal imaging. Left: Venous thrombosis in the sagittal sinus (Top: empty delta sign on contrast CT scan; Bottom: thrombosis in the transverse sinus on MRI) Middle: Other vascular abnormalities (Top: contrast CT showing a vein of Galen malformation in a child with proptosis; ‘tramline’ calcification on CT in a child with Sturge–Weber syndrome) Bottom: Abnormal arteries seen as filling defects (Top: collaterals in moyamoya; Bottom: giant arteriovenous malformation) Right:

**Figure 4:**
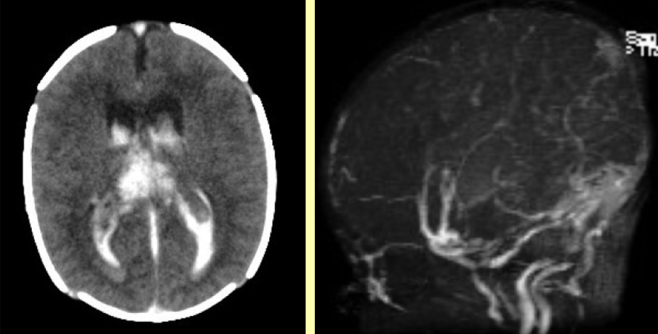
Intraventricular haemorrhage (left) secondary to venous sinus thrombosis (right).

**Figure 5:**
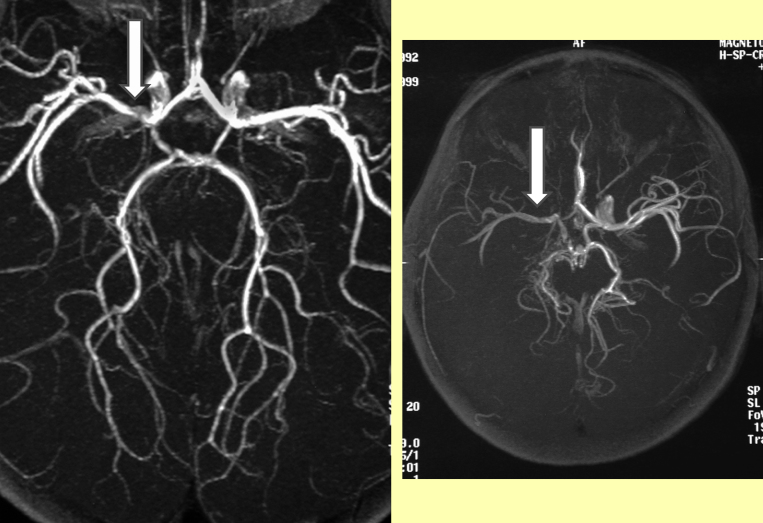
MR angiography of the intracranial vessels Left: focal stenosis typical of focal cerebral arteriopathy of childhood Right: Asymmetry of flow in the intracranial vessels in a child with aortic stenosis suggestive of proximal stenosis or occlusion, e.g. secondary to dissection of the carotid artery, which requires further imaging for diagnosis (see Figure 5).

**Figure 6:**
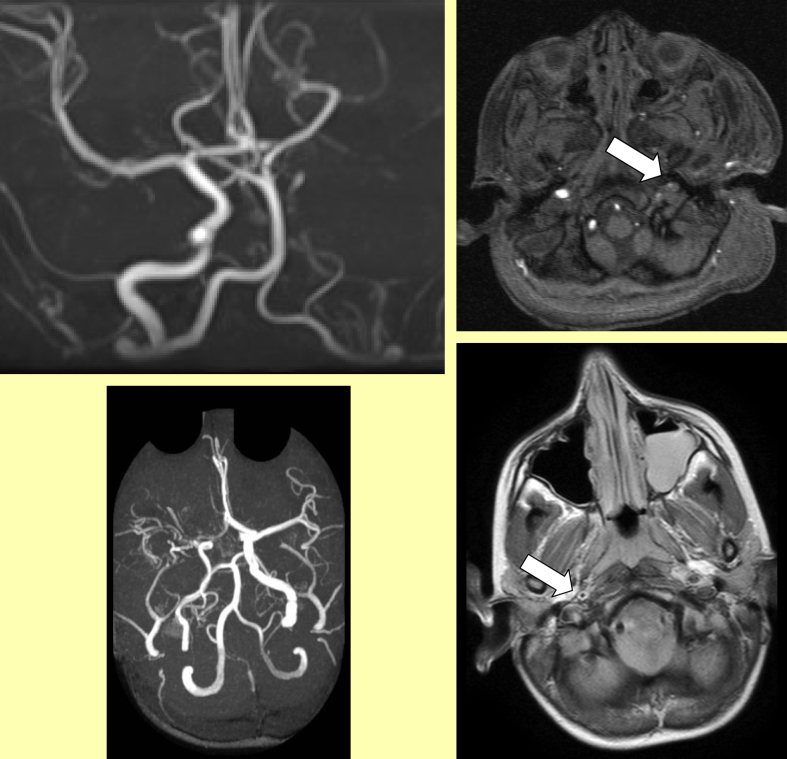
Figure 5: Left: Asymmetry of flow in the cerebral vessels in 2 children with stroke. Right: Fat-saturated T1 MRI of the neck showing carotid occlusion (Top) after trauma from a pencil after a 2 year old fell with it in his mouth (see Figure 11) and the blood in the vessel wall in a child with an apparently spontaneous dissection.

**Figure 7:**
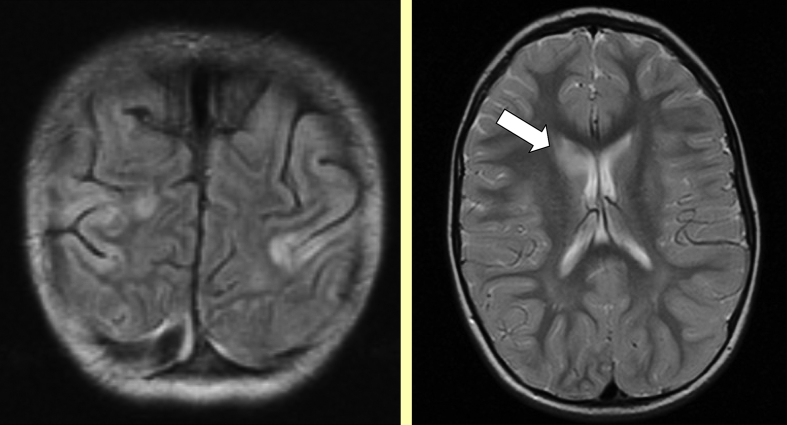
Stroke mimics Left: cortical signal abnormality in an immunosuppressed child with hypertension and posterior reversible encephalopathy syndrome Right: basal ganglia signal abnormality in a child with a recent throat infection who presented with mild unilateral weakness, dystonia and chorea and had a positive antistreptolysin O titre. She did not respond to Penicillin but recovered fully after treatment with steroids.

With limited resources the top priority has to be to undertake imaging at an appropriate interval after the event and ideally in time for intervention if appropriate. Although CT scanning does not show parenchymal infarction reliably within 24 h of an ischaemic stroke, it may be required in the emergency situation to exclude haemorrhage (Figure 1) and may reveal venous sinus thrombosis (Figure 2, Figure 3) and occasionally other vascular abnormalities (Figure 3). CT venography (CTV) and arteriography (CTA) are increasingly used in adults and although the radiation dose is relatively high, this may be the appropriate modality for making the diagnosis quickly in a sick child. MRI scanning, if available together with appropriate anaesthetic cover in view of the length of time required, does also exclude haemorrhage, may reveal venous sinus thrombosis (Figure 3) and may be useful for documenting vascular abnormalities in more detail, including in addition to venography (MRV) (Figure 4) and extra- and intracranial arteriography (MRA) (Figure 5), sequences for fat-saturated T1 MRI of the neck (FS-T1) (Figure 6). If there is no obvious arterial disorder involving the intracranial vessels on MRA (Figure 5), venous sinus thrombosis should be investigated either with CTV or MRV (Figure 4) and dissection in the neck vessels should be excluded, if necessary with conventional arteriography if neck FS-T1 and MRA (Figure 6) are not conclusive. MRI is also the modality of choice for diagnosing stroke mimics (Figure 7).

### Neurophysiology (Table 1)

**Figure 8:**
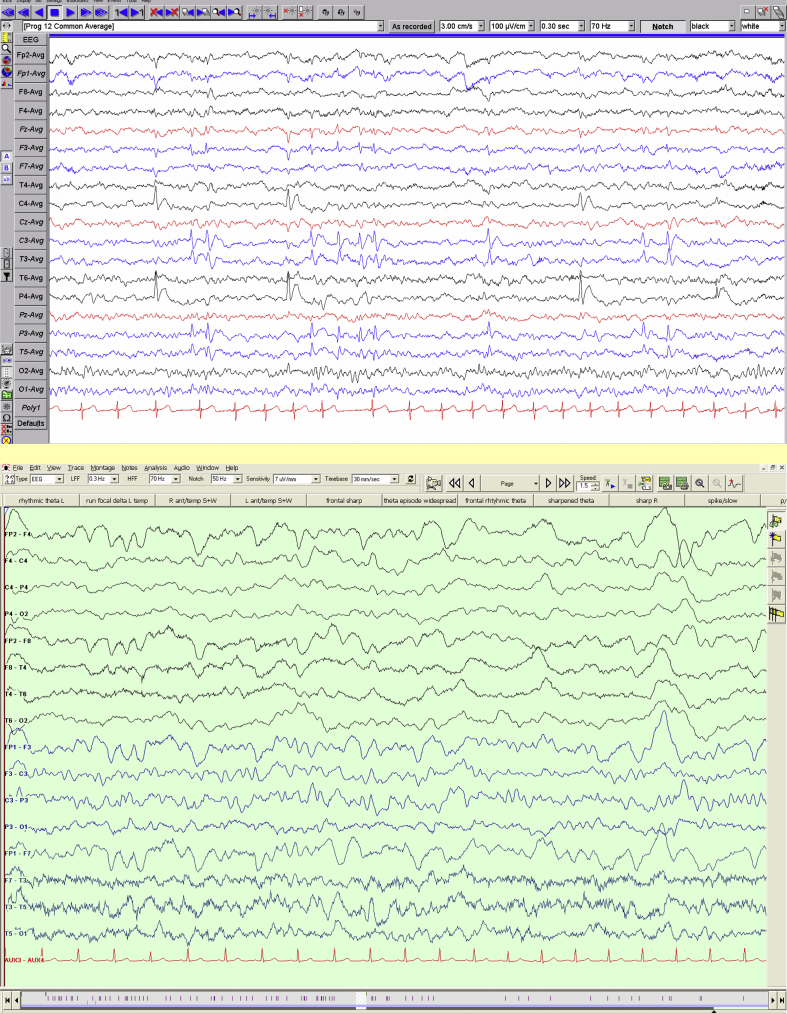
Electroencephalography Top: bilateral independent discharges, occurring in typical doublets (and a quadruplet) on the left, typical of benign Rolandic epilepsy, in a child presenting with a persistent, but ultimately reversible, hemiparesis. Bottom: unilateral slowing on EEG in a child with familial hemiplegic migraine and a calcium channel gene mutation.

Electroencephalography can be useful in demonstrating that focal signs are epileptic or migrainous in origin (Figure 8).

### Echocardiography (Table 1)

**Figure 9:**
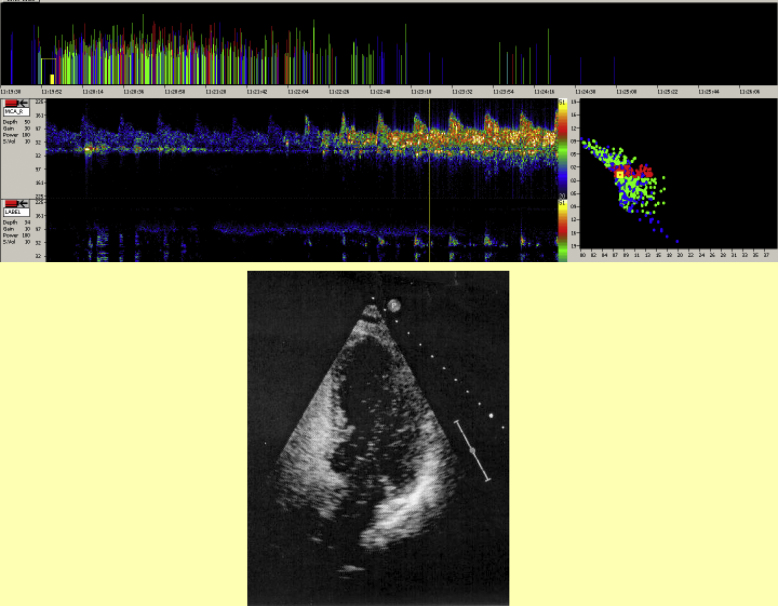
Excluding a right to left shunt. Top: Positive Patent Foramen Ovale study using a DWL EmboDop transcranial Doppler system that uses multifrequency and multiple depths simultaneously. The upper window shows the output from a 2MHz probe at 55mm depth and the lower window is 2MHz at 38mm depth (the reference gate) which in this case demonstrates that the embolic shower that is seen at 55mm is not an artefact as it is not shown simultaneously at the reference gate. Bottom: Echocardiogram during bubble contrast showing passage of bubbles from the right to the left atrium.

All children with stroke, whether or not they have another underlying condition, should have a transthoracic echocardiogram, ideally with bubble contrast during a Valsalva manoeuvre to detect a significant right to left shunt at atrial level (Figure 9). Poor ventricular function is at least as common as patent foramen ovale on echocardiography. The role of bubble contrast transcranial Doppler (Figure 9) in detecting minor degrees of shunting has not yet been established and the need to close any patent foramen ovale found is currently very controversial.

### Laboratory (Table 1)

From the point of view of the laboratory diagnosis it is important to examine the red blood cell indices, since a substantial proportion of young children with cerebrovascular disease (particularly venous sinus thrombosis) have iron deficiency which can be proven in most cases with a ferritin or, if necessary, more detailed iron studies. There is now substantial evidence linking prothrombotic disorders to childhood stroke, but extensive investigation is expensive. In some circumstances it may be appropriate to limit the investigation to the tests which will make a difference to management, for example excluding prothrombin 20210, which appears to increase the risk of recurrence in venous sinus thrombosis.

## Management (Table 1)

**Figure 10:**
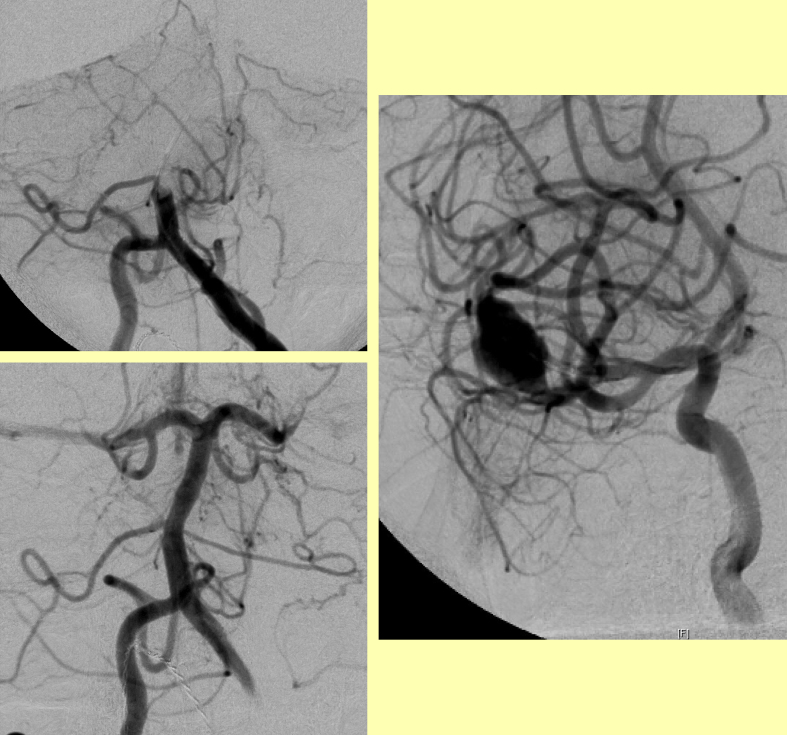
Candidates for interventional neuroradiology. Left: Basilar occlusion (Top) in a child who remained in coma for 11 h; after intra-arterial mechanical thrombectomy and thrombolysis he regained consciousness and made a good recovery Right: Dissecting aneurysm of the middle cerebral artery in a child who developed a severe headache whilst playing the trumpet and had a subarachnoid haemorrhage. He had a small subclinical ischaemic stroke after a stenting procedure but the risk of recurrent haemorrhage (at least 1% per year) was reduced.

**Figure 11:**
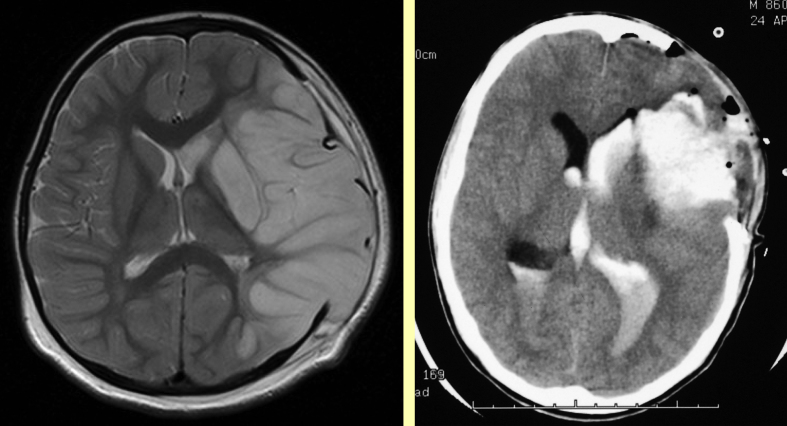
Surgical decompression in ischaemic (left) and haemorrhagic (right) stroke.

If there is any reduction in conscious level the child requires emergency transfer to a centre in which emergency intervention neuroradiology and neurosurgery can be. Rapid imaging such as CT scanning can be performed while transportation is organized but neuroimaging should not delay transfer. It is very important that principles of good emergency management of a sick child are followed, with attention to management of the airway, circulation and any seizures. There are no randomized controlled trials of emergency management of stroke in childhood and thrombolysis is not recommended for children in view of the risk of bleeding, although the American Stroke Association guidelines comment that there was no consensus for teenagers and there have been several case reports of successful thrombolysis, particular for posterior circulation occlusion (Figure 10) where there is a 12-h time window. Occasional children with a high risk of recurrent stroke may be suitable for interventional neuroradiological procedures (Figure 10), such as coils or stents, as well as vascular neurosurgery e.g. clipping of an aneurysm or stereotactic radiotherapy, e.g. to obliterate an arteriovenous malformation. Surgical decompression may be life-saving if the patient is deeply unconscious (Figure 11).

For arterial ischaemic stroke, in view of the data from adults and the low risk of haemorrhage and of Reye’s syndrome, most physicians give Aspirin at a dose of 5 mg/kg/day in the acute situation and continue this at the same or a dose as low as 1 mg/kg/day. Recent cohort studies suggest that Aspirin prophylaxis may have been associated with reduction in the risk of recurrence, at least for anterior stroke, but there have been no randomized controlled trials. Many physicians would anticoagulate older children with venous sinus thrombosis, as the adult randomized controlled trials showed benefit in terms of mortality and morbidity and the risk of haemorrhage is low. Some physicians would also anticoagulate children with proven extracranial dissection but not those with intracranial dissection because of the risk of subarachnoid haemorrhage.

Although prothrombotic disorders do appear to be associated with stroke risk and probably with the risk of recurrence, investigation of individual risk factors has not yet led to secondary prevention. It is important to exclude or treat iron deficiency and to ensure all children have a healthy diet full of fruit and vegetables, a reduced fat intake and plenty of exercise. Long term low dose aspirin is a relatively low risk strategy which many physicians use to attempt to prevent secondary arterial ischaemic stroke. For children who have had a venous sinus thrombosis anticoagulation is usually continued for 3–6 months and then discontinued except in high risk situations such as recurrence of nephrotic syndrome or exacerbation of inflammatory bowel disease. However children with the prothrombin 20210 mutation are at high risk of recurrence and it is reasonable to undertake this test and to consider long term anticoagulation in children who have had venous sinus thrombosis in this context without any other triggers.

### Rehabilitation and reintegration into school

Some children make a very rapid recovery from stroke while others have considerable residual disability. Early rehabilitation by a skilled team can make a big difference to the long term outcome. Children may need an educational statement on return to school, and they and their families need considerable support.

**Practice points**

• Neuroimaging, usually requiring venous as well as intracranial and extracranial arterial vascular sequences and diffusion-weighted imaging, should be performed as early as possible to distinguish aetiology of childhood stroke (Table 1).
• EEG may be useful in the distinction of cerebrovascular disease from epilepsy and migraine.
• Echocardiography may allow diagnosis of patent foramen ovale as well as poor ventricular function but there is considerable controversy about closure and this is not recommended until randomized controlled trials have reported.
• In units with appropriate experience, unconscious children with stroke may occasionally be offered thrombolysis or surgical decompression which may be life-saving.
• Strategies for individual children should be considered carefully in order to reduce the high risk of recurrence for haemorrhagic and ischaemic stroke.

## Figures and Tables

**Table 1:** Differential diagnosis, investigation and management in the child with suspected stroke

**Aetiology**	**Clinical/laboratory/neuroradiological features**	**Specific treatments to be considered**
*Space-occupying mass*	Focal signs, seizures, deteriorating level of consciousness	Surgical opinion
-Spontaneous intracerebral haemorrhage	Sudden onset, obvious on plain CT, may be secondary to VST so CTV/MRV, distinction between aneurysm and AVM may require MR + conventional arteriography	Surgical opinion ?decompression, exclude bleeding diastheses, polycystic kidneys and other genetic causes of AVM or aneurysm
-Ischaemic stroke- anterior circulation e.g. large hemispheric	Preceding transient ischaemic attacks in some cases, at <24 h may be subtle changes on CT but MRI often required	Unusual to present <4.5 h so NOT thrombolysis. ?Surgical decompression if deeply unconscious
-Ischaemic stroke- posterior circulation e.g. cerebellar (with hydrocephalus) or brainstem	Preceding transient ischaemic attacks in some cases, at <24 h may be subtle changes on CT but MRI often required	Consider thrombolysis in teenager at <12 h Surgical opinion drainage/decompression
-Tumour	Preceding headache and other symptoms & signs, CT+/-MRI	Surgical opinion
-Cerebral abscess	Fever, Obvious on contrast CT	Antibiotics including cover for anaerobes
*Venous sinus thrombosis*	Focal signs, seizures, deteriorating level of consciousness, haemorrhage or ischaemia or normal CT; needs CTV or MRV	Anticoagulation, exclude prothrombotic disorders especially Prothrombin 20210
*Accidental head injury*	History of head injury	
-Extradural or intracerebral haematoma	Obvious on plain CT	Surgical opinion
-Extracranial dissection	Fat-saturated T1 MRI of neck shows blood in vessel wall	Consider anticoagulation; may be suitable for interventional neuroradiology
-Intracranial dissection	Double lumen may be demonstrated on MRA or conventional arteriography	Anticoagulation contraindicated; may be suitable for interventional neuroradiology if haemorrhage in view of recurrence risk
-Diffuse brain oedema	Exclude venous sinus thrombosis on CTV or MRV	Surgical opinion decompression
*Non-accidental injury*	Retinal hemorrhages on funduscopy, bruises, fractures	Child protection
-Subdural haemorrhage/effusion		Surgery opinion
-Intracerebral haemorrhage		Surgery opinion
-Hemispheric ischaemia, diffuse brain oedema	Exclude secondary VST	Surgical opinion decompression
*Infections*		
-Meningitis	Fever, nuchal rigidity, purulent CSF, PCR	
-*Streptococcus pneumoniae, Hemophilus influenza, Neisseria meningitides*	May have AIS or VST	3rd generation Cephalosporin, aspirin, anticoagulation for VST, ensure adequate iron, folate, B6 intake
-*Mycobacterium Tuberculosis*	Hydrocephalus, cerebrovascular involvement, PCR	Anti-tuberculous therapy, aspirin
-Chickenpox	History in previous year, MRA shows basal ganglia stroke and focal cerebral arteriopathy of childhood	Aspirin, ensure adequate iron, folate, B6 intake
-Upper respiratory tract infection	Recent history, MRA typically shows basal ganglia stroke and focal cerebral arteriopathy of childhood	Aspirin, ensure adequate iron, folate, B6 intake
-Borrelia (Lyme disease)	Recent history, rising serum or CSF titres, MRA typically shows basal ganglia stroke & focal cerebral arteriopathy of childhood	3rd generation Cephalosporin, Aspirin, ensure adequate iron, folate, B6 intake
*-*Enterovirus	Recent history, rising serum or CSF titres, MRA typically shows basal ganglia stroke & focal cerebral arteriopathy of childhood	Aspirin, ensure adequate iron, folate, B6 intake
*Human immunodeficiency virus*	Systemic illness, may have aneurysms, focal cerebral arteriopathy of childhood or moyamoya	Antiretrovirals, ensure adequate iron, folate, B6 intake
-Post-Streptococcal hemiparesis and dystonia/chorea	Throat infections, Positive ASOT, MRI may show signal change not typical for ischaemia	Penicillin as for typical Sydenham’s
Acute disseminated encephalomyelitis (ADEM)	Demyelination on MRI, may have had infection	Corticosteroids, IVIG
Congenital heart disease	Exclude VST, dissection, moyamoya, aneurysm, embolus	Discuss with cardiologists
Sickle cell disease	Exclude VST, PRES, focal cerebral arteriopathy of childhood, dissection, moyamoya, aneurysm, embolus through PFO	Exchange transfusion-very slowly Appropriate management stroke syndrome
Other anaemias including iron deficiency	Exclude VST, PRES, focal cerebral arteriopathy of childhood, dissection, moyamoya, aneurysm, embolus through PFO	Appropriate management of anaemia and stroke syndrome; care with transfusion
Haemolytic-uraemic syndrome	Anaemia, jaundice, Burr cells on blood film, AIS, VST or PRES	Dialysis; Appropriate management of anaemia and stroke syndrome
Nephrotic syndrome	Typically VST	Anticoagulate acutely and in relapse
Inflammatory bowel disease	VST, PRES, focal cerebral arteriopathy of childhood	VST Anticoagulate acutely and in relapse
Leukaemia	VST, PRES, focal cerebral arteriopathy of childhood	VST Anticoagulate acutely and in relapse
Hypoglycaemia	Encephalopathic, hemiparesis, seizures	Glucose
Epilepsy	Subtle seizures, EEG may show e.g. Rolandic spikes (Figure 9)	Consider anticonvulsants
Hypertensive encephalopathy	Preceded by visual symptoms & seizures, macular star, CT may show subtle changes but MRI (DWI) shows PRES	Slow reduction blood pressure
Migraine e.g. hemiplegic	Family history, headache, EEG shows unilateral slowing (Figure 9)	May respond to calcium channel blockers, phenytoin or acetazolamide
*Metabolic conditions*		
-Ornithine transcarbamylase deficiency	Unilateral cerebral oedema; High ammonia	
-Mitochondrial	MRI: parieto-occipital lesions not typical of AIS; High lactate	Arginine
Moyamoya	Preceding transient ischaemic attacks in some cases, may be a family history or clues to an underlying diagnosis	Revascularization
Lacunar stroke with no obvious precipitant and normal vascular imaging	Exclude PFO using transoesophageal ECHO; role of bubble TCD not established	Long term aspirin; consider closure of PFO after RCTs have evaluated

AIS: arterial ischaemic stroke; VST: venous sinus thrombosis; PRES: posterior reversible encephalopathy syndrome; AVM arteriovenous malformation.

CT: computed tomography; CTV: CT venography; MRI: magnetic resonance imaging; MRV: MR venography; MRA: MR arteriography; DWI: diffusion-weighted imaging.

ASOT: antistreptolysin O titre; PCR: polymerase chain reaction; EEG: electroencephalography; ECHO echocardiography; TCD: transcranial Doppler.
